# Neuronal tangential migration from *Nkx2.1*-positive hypothalamus

**DOI:** 10.1007/s00429-020-02163-x

**Published:** 2020-11-03

**Authors:** Raquel Murcia-Ramón, Verónica Company, Iris Juárez-Leal, Abraham Andreu-Cervera, Francisca Almagro-García, Salvador Martínez, Diego Echevarría, Eduardo Puelles

**Affiliations:** Instituto de Neurociencias de Alicante, Universidad Miguel Hernández-CSIC, 03550 Sant Joan d’Alacant, Alicante Spain

**Keywords:** *Nkx2.1*, Hypothalamus, Tangential migration, Zona incerta, Reticular formation

## Abstract

During the development of the central nervous system, the immature neurons suffer different migration processes. It is well known that *Nkx2.1*-positive ventricular layer give rise to critical tangential migrations into different regions of the developing forebrain. Our aim was to study this phenomenon in the hypothalamic region. With this purpose, we used a transgenic mouse line that expresses the *tdTomato* reporter driven by the promotor of *Nkx2.1*. Analysing the *Nkx2.1*-positive derivatives at E18.5, we found neural contributions to the prethalamic region, mainly in the zona incerta and in the mes-diencephalic tegmental region. We studied the developing hypothalamus along the embryonic period. From E10.5 we detected that the *Nkx2.1* expression domain was narrower than the reporter distribution. Therefore, the *Nkx2.1* expression fades in a great number of the early-born neurons from the *Nkx2.1*-positive territory. At the most caudal positive part, we detected a thin stream of positive neurons migrating caudally into the mes-diencephalic tegmental region using time-lapse experiments on open neural tube explants. Late in development, we found a second migratory stream into the prethalamic territory. All these tangentially migrated neurons developed a gabaergic phenotype. In summary, we have described the contribution of interneurons from the *Nkx2.1*-positive hypothalamic territory into two different rostrocaudal territories: the mes-diencephalic reticular formation through a caudal tangential migration and the prethalamic zona incerta complex through a dorsocaudal tangential migration.

**Electronic supplementary material:**

The online version of this article (10.1007/s00429-020-02163-x) contains supplementary material, which is available to authorized users.

## Introduction

In embryonic development, the neural stem cells, that give rise to neurons and glia, are known as neuroblasts (Nieuwenhuys et al. 2008). These cells produce all the different neuronal subtypes that constitute a mature brain. To trigger the different specification programs, these neuroblasts must know their exact position along the axes of the developing neural tube. In the antero-posterior axis, two regions, the isthmus and the zona limitans intrathalamica, have been identified as secondary organizer centers (Echevarría et al. 2003). In the dorso-ventral axis, two regions, the floor and the roof plates, has also been located. The roof plate contains the anterior neural ridge, a specialized part, with dorsalizing effects on the forebrain including the hypothalamus (Puelles and Rubenstein 2015). These organizers are groups of cells located in key regions of the neural tube that secrete proteins. The concentration gradients of these protein substances, known as morphogens, supply the needed positional information to the surrounding neuroblasts. This information will, therefore, prompt specific differentiation programs that will direct the specification of the different territories and neuronal phenotypes (Anderson and Stern 2016). Once the neuroblasts are committed to their neural destiny, the young neurons must migrate to occupy their final location in the brain. All of them suffer a radial migration, from ventricular to the mantle layer. In this process, they use the radial glia scaffold. After this centrifugal movement, some of the immature neurons start a free migration along the rostrocaudal and/or dorsoventral axes named tangential migration (Moffat et al. 2015). This process is mediated by long and short distance guidance cues that allow the neurons to find the correct direction towards their final destination (Hatanaka et al. 2016).

The *Nkx2.1* gene belongs to the Nkx transcription factors family (Guazzi et al. 1990). They have been involved in several differentiation genetic cascades of not only neuronal and glial cells but other cellular subtypes in different organs (liver, lungs, thyroid gland, etc., Minoo et al. 1995; Briscoe et al. 1999; Watada et al. 2000, 2003; Prakash et al. 2009; Cai et al. 2010). *Nkx2.1* is expressed during brain development in two domains located in the secondary prosencephalon. One sited in the medial ganglionic eminence and preoptic area, part of the subpallial territory of the telencephalic vesicle (Marin et al. 2000; Pleasure et al. 2000; Puelles et al. 2001). The other covers most of the basal hypothalamic region, leaving the retromamillar area negative (see Figs. 5h and 1d in Morales-Delgado et al. 2011, 2014, respectively; see Figs. 8.9 and 8.10D in Puelles et al. 2012).

In the subpallial positive territory, centered in the medial ganglionic eminence, the *Nkx2.1* domain will give rise, from ventricular to the pial surface, to the bed nucleus of the stria terminal (laterocentral), the external and ventral pallidum, the pallidal olfactory tuberculum and the central amygdaloid nucleus that belongs to the pallidal amygdala (Allen Developing Mouse Brain Atlas, online since 2009; www.developingmouse.brain-map.org; Puelles et al. 2000,2016; García-López et al. 2008). From all these neuronal populations, only the external pallidus will retain the *Nkx2.1* expression up to adulthood (Marin et al. 2000). On top of all these derivatives, the medial ganglionic eminence will also give rise to the gabaergic interneurons that will populate the cerebral cortex after a long tangential migration (Sussel et al. 1999; Marin et al. 2000). These neurons will switch off the *Nkx2.1* expression as soon as they start their migration process described thanks to the use of genetic reporter tools (Marin et al. 2000).

In the hypothalamic territory, the *Nkx2.1* derivatives actively contribute to the terminal and peduncular hypothalamic prosomeres (Puelles et al. 2012; Puelles and Rubenstein 2015; Puelles 2019). Populations as the mamillary nucleus (see Puelles and Rubenstein 2015 for the spelling of mamillary), arcuate nucleus, some of the ventromedial and dorsomedial subnuclei and the lateral hypothalamic presents *Nkx2.1*-positive neurons. Other hypothalamic nuclei lose the expression of this transcription factor after differentiation (Nakamura et al. 2001). Several tangential migrations have been described within and to the basal hypothalamus. Contributions to the arcuate nucleus, ventromedial nucleus, ventral premamillar nucleus, and some of the migrated were found to be peptidergic neurons have been discovered (Morales-Delgado et al. 2011, 2014; Díaz et al. 2015; Alvarez-Bolado 2019).

In contrast to the subpallial domain, no tangential migration from the basal hypothalamic regions into surrounding territories has been described. The aim of our work has been to analyze the *Nkx2.1* hypothalamic derivatives destiny, using a transgenic mouse line that express the *tdTomato* reporter driven by the promotor of *Nkx2.1*. Thus, we have been able to identify, for the first time, two tangential migrations from the hypothalamus into the alar prethalamus (p3) and into the basal diencephalic territory (up to p1).

## Results

### Final distribution of Nkx2.1 hypothalamic derivatives

As an initial point in our research, we decided to describe the location of the neuronal populations generated in the *Nkx2.1*-positive hypothalamic territory in late embryonic development. Since in the subpallial territory the tangentially migrated neurons with a *Nkx2.1*-positive origin switch off their expression, we used a reporter line (*Nkx2.1*^*cre/*+^*; tdTomato*^*flox/*+^) to unveil the final destination of all the *Nkx2.1*-positive hypothalamic neuronal derivatives.

**Fig. 1:**
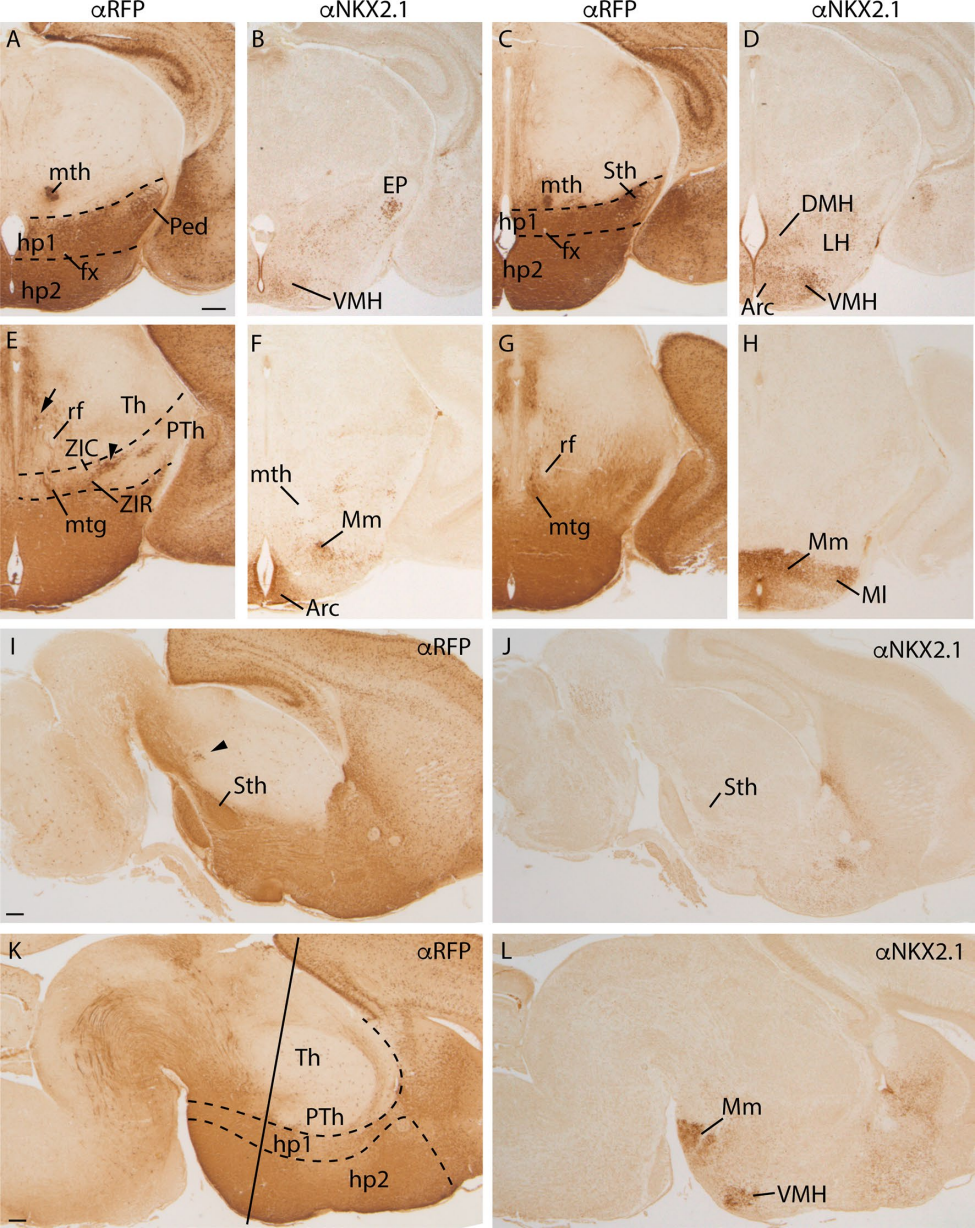
Frontal sections of *Nkx2.1*^*cre/*+^*; tdTomato*^*flox/*+^ E18.5 embryos, labelled against RFP (**a, c, e, g**) and NKX2.1 (**b, d, f, h**) from dorsal to ventral. Sagittal sections of *Nkx2.1*^*cre/*+^*; tdTomato*^*flox/*+^ E18.5 embryo, labelled against RFP (**i, k**) and NKX2.1 (**j, l**). The arrowhead shows the positive RFP derivatives located in Zona incerta (**e, i**). The arrows point to the positive derivatives sited in the periventricular mes-diencephalic basal plate (**e**). Note the migrated interneurons that colonized the entire cortex with an strong RFP labelling. The most medial part of the entopeduncular nucleus is also RFP positive and retains NKX2.1 presence (**b**) meanwhile the subthalamic nucleus being RFP positive do not contain NKX2.1 protein (**d, j**). The RFP labelling observed in the alar hypothalamus correspond with positive fascicles originated in the basal hypothalamus. The dashed lines indicate the limit between the hypothalamic prosomeres hp1 and hp2. The line in K indicates the section plane of the frontal sections. *Arc* arcuate nucleus, *DMH* dorsomedial hypothalamic nucleus, *EP* entopeduncular nucleus, *fx* fornix tract, *hp1* hypothalamic prosomere 1 (peduncular hypothalamus), *hp2* hypothalamic prosomere3 2 (terminal hypothalamus), *LH* lateral hypothalamic nucleus, *Mm* medial mamillary nucleus, *Ml* lateral mamillary nucleus, *mtg* mamillo tegmental tract, *mth* mamillo thalamic tract, *Ped* cerebral peduncle, *Pth* prethalamus, *rf* retroflex tract, *Sth* Subthalamic nucleus, *Tb* tuberal region, *Th* thalamus, *VMH* ventromedial hypothalamic nucleus, *ZIC* zona incerta caudal, *ZIR* zona incerta rostral. Scale bar = 200 μm

In an E18.5 mouse embryo, we analyzed the red fluorescent protein (RFP) and NKX2.1 protein distribution. The tomato protein (Fig. 1a, c, e, g, i, k) was localized in all the basal hypothalamic domain, including the tuberal and mamillary regions. Among all this general labelling, a strong staining of the subthalamic nucleus (Fig. 1c, e, i), two positive groups of neurons in the caudal portion of the zona incerta (arrowhead in Fig. 1g, i; Puelles et al. 2020) and scattered positive cells in the periventricular mes-diencephalic basal plate (arrow on Fig. 1e) draw our attention. The axonal tracts originated in the hypothalamic region also appeared labelled, as the mamillothalamic tract (Fig. 1a, c) and the mamillotegmental tract (Fig. 1e, g), whereas the retroflex tract (Fig. 1e, g) was negative as it is generated in a *Nkx2.1* negative territory.

The NKX2.1 protein was detected (Fig. 1b, d, f, h, j, l) in the ventricular layer and in several hypothalamic nuclei located in the mantle layer that maintained the expression of the gene after the differentiation process. The ventromedial hypothalamic nucleus (VMH) and the dorsomedial hypothalamic nucleus (DMH; Fig. 1b, d) presented medium density of positive neurons. However, the arcuate nucleus (Arc), the medial mamillary nucleus (Mm) and the lateral mamillary nucleus (Ml) displayed a high density of labelled neurons (Fig. 1f, h, l).

### Early detection of Nkx2.1 pattern compared with its derivatives

**Fig. 2:**
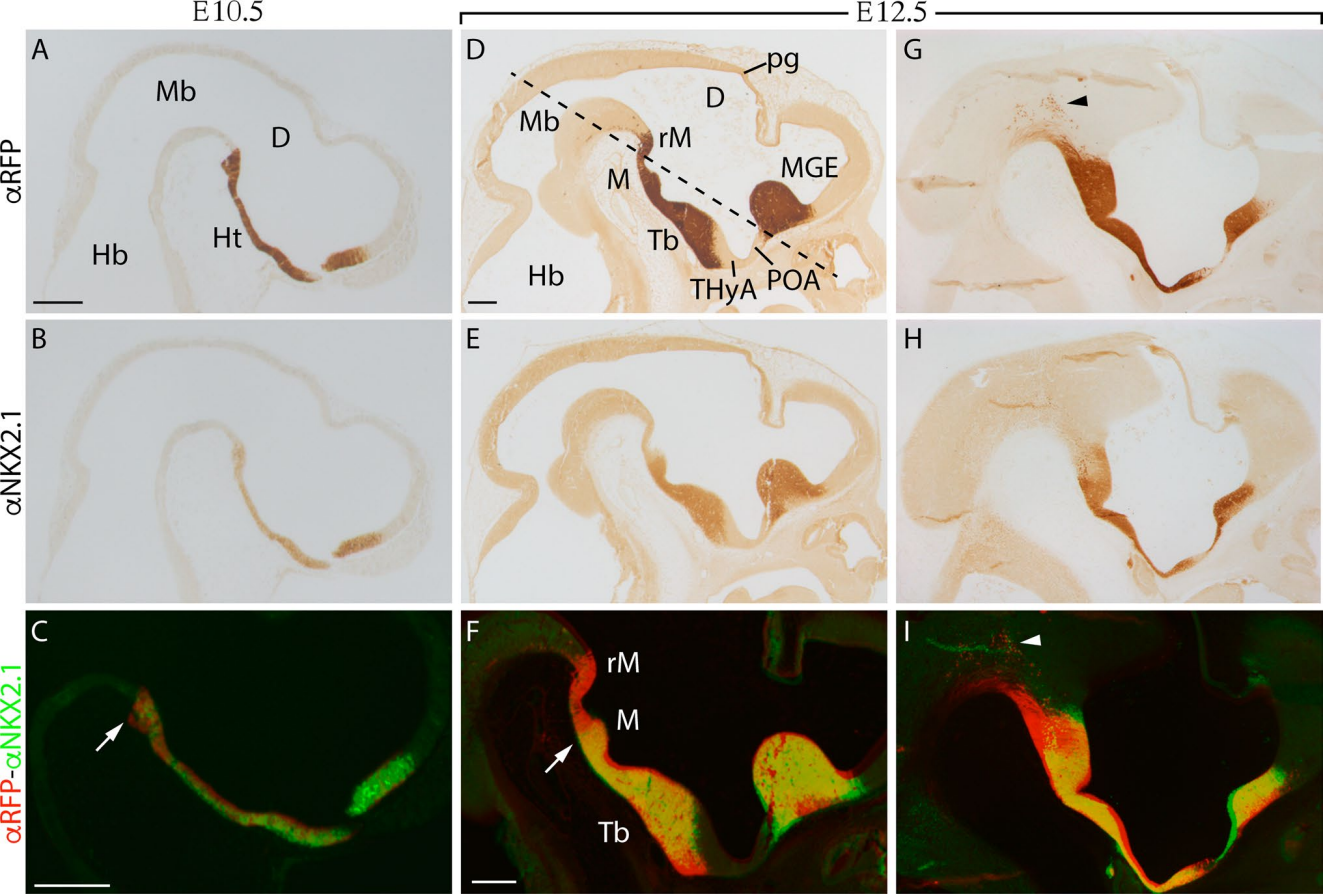
Sagittal sections of *Nkx2.1*^*cre/*+^*; tdTomato*^*flox/*+^ embryos. **a, b** E10.5 labelled against RFP and NKX2.1 respectively. **c** Merge of the previous images. The arrow points out the caudal end territory of NKX2.1 that coincides with the RFP expression pattern. **d, e, g, h** medial and lateral E12.5 slices labelled against RFP and NKX2.1 respectively. In D, we identified between the two NKX2.1-positive domains the negative preoptic area and the negative alar terminal hypothalamus. **f, i** Merge of D and E; and G and H, respectively. The arrow points out the caudal limit of the NKX2.1 labelling, which now is narrower than the RFP domain. This indicates that the *Nkx2.1* expression domain is reduced along time. The arrow head indicates some postmitotic tomato-positive neurons tangentially migrating caudally. The dashed line indicates the section plane of Fig. 3. At the bottom right, we have the most rostral part and in the upper and upper right part we have the dorsal part. *D* diencephalon, *Hb* hindbrain, *Ht* hypothalamus, *M* mamillary region, *Mb* midbrain, *MGE* medial ganglionic eminence, *pg* pineal gland, *POA* preoptic area, *rM* retromamillary region; Tb, tuberal region; THyA, alar terminal hypothalamus. Scale bar = 200 μm

Due to the sharp divergence between the NKX2.1 protein distribution and the derivatives location in late embryonic stages, we aimed to unveil the development of this discrepancy. We studied the tomato protein-positive territory (Fig. 2a, d, g) compared with the NKX2.1 protein distribution (Fig. 2b, e, h) in early embryos. At E10.5, the coincidence between both protein distributions is complete. Note that the caudal NKX2.1 and RFP limit coincided clearly (white arrow in Fig. 2c). At E12.5, we observed the evolution of these territories. In a mid-sagittal section, the distribution of both proteins is similar, but the caudal limit of NKX2.1 is located in the mamillary eminence (Fig. 2e; white arrow in Fig. 2f) while the territory of the tomato protein is maintained in the retromamillary territory (Fig. 2d, f), which means that *Nkx2.1* is silenced in the ventricular layer of the most caudal hypothalamic regions. Surprisingly, in the lateral areas, labelled against RFP, we observed some post-mitotic *Nkx2.1* derivatives located caudally to the hypothalamic region. This would imply that they have migrated tangentially caudalwards from the hypothalamic territory (arrowhead in Fig. 2g, i). These cells were not detectable with the NKX2.1 antibody (Fig. 2H).

### Early tangential migratory stream

**Fig. 3:**
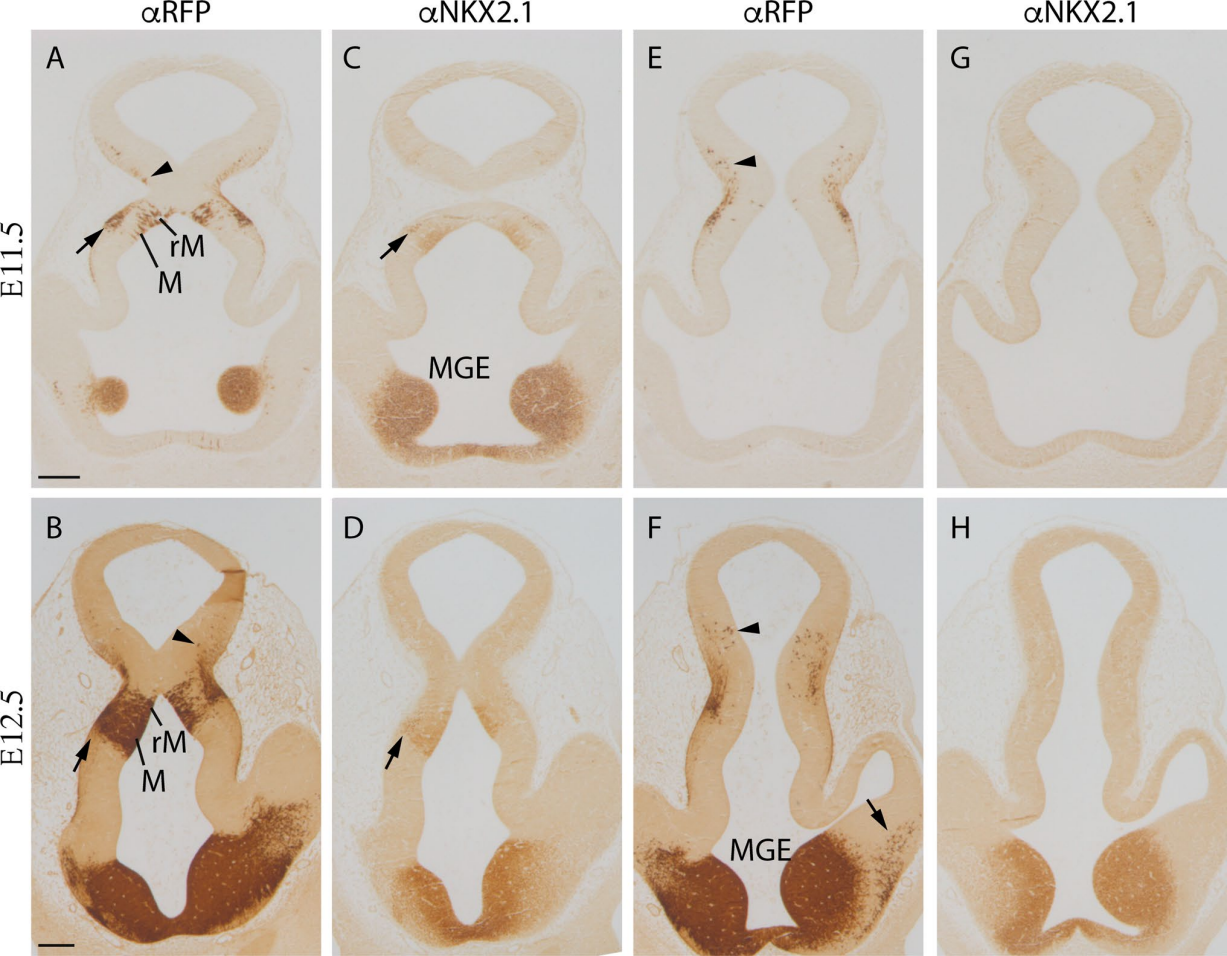
Frontal sections of *Nkx2.1*^*cre/*+^*; tdTomato*^*flox/*+^embryos following the plane of section indicated in Fig. 2d from ventral (**a–d**) to dorsal (E–H). **a, e** E11.5 labelled against RFP. **c, g** E11.5 labelled against NKX2.1. **b, f** E12.5 labelled against RFP. **d, h** E12.5 labelled against NKX2.1. The arrowhead points out the tomato positive neurons migrating tangentially from the mamillary territory and the arrow points out the migration of interneurons from the MGE into the cortex. *M* mamillary region, *MGE* medial ganglionic eminence, *rM* retromamillar region. Scale bar = 200 μm

Next, we aimed to describe this migration process using transversal plane sections (see dashed line in Fig. 2d) to study in the same section the *Nkx2.1*-positive ventricular domain and the Tomato-positive migrating neurons. At E11.5, we already observed some positive tomato neurons migrating tangentially from the retromamillary region (arrowheads in Fig. 3a and e). Other neurons, still *Nkx2.1* positive, were also observed migrating radially from the ventricle to the pial surface (arrow in Fig. 3a and c). The distant migrating neurons (arrowhead in Fig. 3e) are no longer positive for NKX2.1 (Fig. 3g).

At E12.5, the same migration phenomenon was observed in the retromamillary region but the number of neurons increased (arrowheads in Fig. 3b and f). In the mamillary region, NKX2.1 and RFP-positive neurons migrated radially (arrow in Fig. 3b and d). As previously observed, the distant migrated neurons are negative for NKX2.1 (Fig. 3h). We could also detect the well-known migration of interneurons from the medial ganglionic eminence into the cortex (arrow in Fig. 3f).

**Fig. 4:**
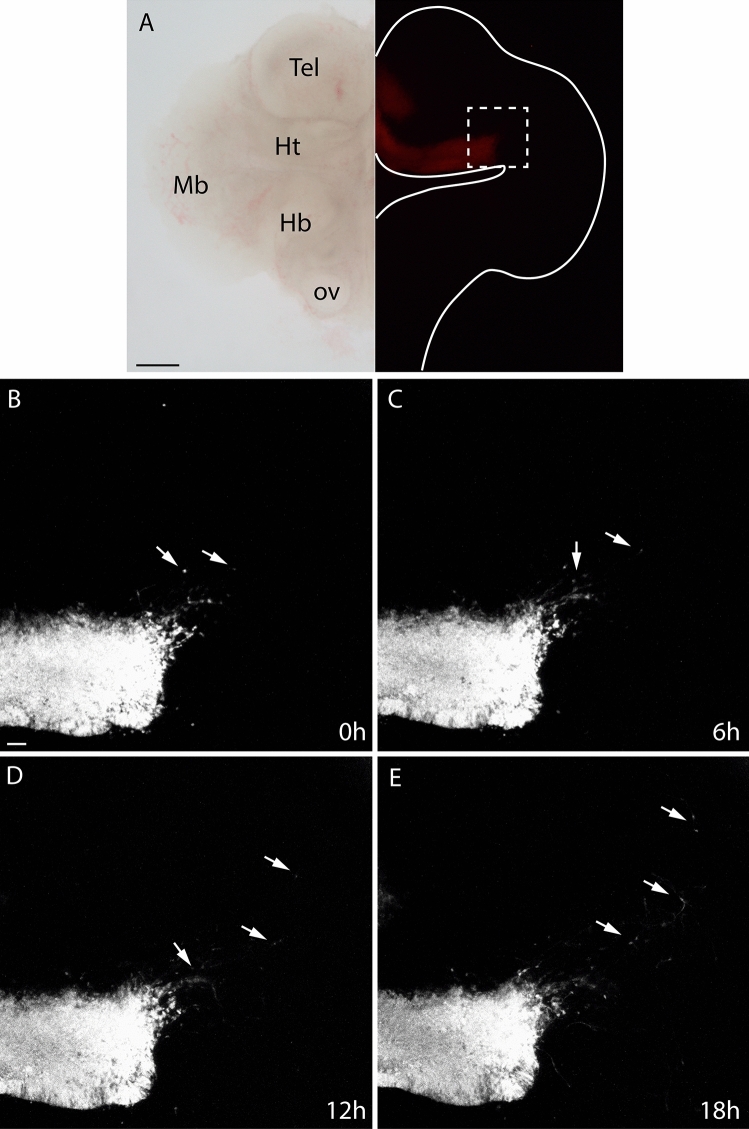
Organotypic neural tissue culture (ONTC) explant of *Nkx2.1*^*cre/*+^; *tdTomato*^*flox/*+^ embryo at E10.5. The dashed line delimits the magnifications. **a** Brightfield of an ONTC (left) and the same ONTC under fluorescence microscope (in red the tomato-positive domain). The line delimits the explant and the dashed line shows the magnification zone of the following images. The explant was incubated during 18 h and the fluorescence was recorded every 20 min. The images show different acquisition times: 0 h (**b**), 6 h (**c**), 12 h (**d**), and 18 h (**e**). The arrows point some examples of these migrating neurons. *Tel* telencephalon, *Ht* hypothalamus, *Mb* midbrain, *Hb* Hindbrain, *ov* otic vesicle. Scale bar = 200 μm in **a** and 20 μm in **b–e**

To complete our previous descriptive analysis and prove this migratory process, we did a real-time image acquisition of the migration. For this purpose, we performed a time-lapse experiment of organotypic neural tissue culture (ONTC) of an E10.5 transgenic embryo. In this sample, we were able to follow the migrating neurons due to the red fluorescence of the *Nkx2.1*-positive cells. An ONTC bright field micrography is displayed in Fig. 4a (left) and its autofluorescence due to the tomato protein can be observed in Fig. 4a (right).

The explant was incubated for 18 h under an inverted confocal microscopy and the red autofluorescence was recorded every 20 min. Then, the obtained images were composed, and a video was made (ESM1). The time-lapse video proved that the red fluorescence neurons, which previously expressed *Nkx2.1* at caudal territories, migrated tangentially along time towards this caudal territory during the recording time assay (arrows in Fig. 4b–e).

The neurons that participate in this early migratory event are finally localized in the mes-diencephalic reticular formation (arrow in Fig. 1e).

### Late tangential migratory stream

Once we described the timing, route and final destination of the early migration observed, we aimed to study the colonization by *Nkx2.1* derivatives of the prethalamic territory observed.

**Fig. 5:**
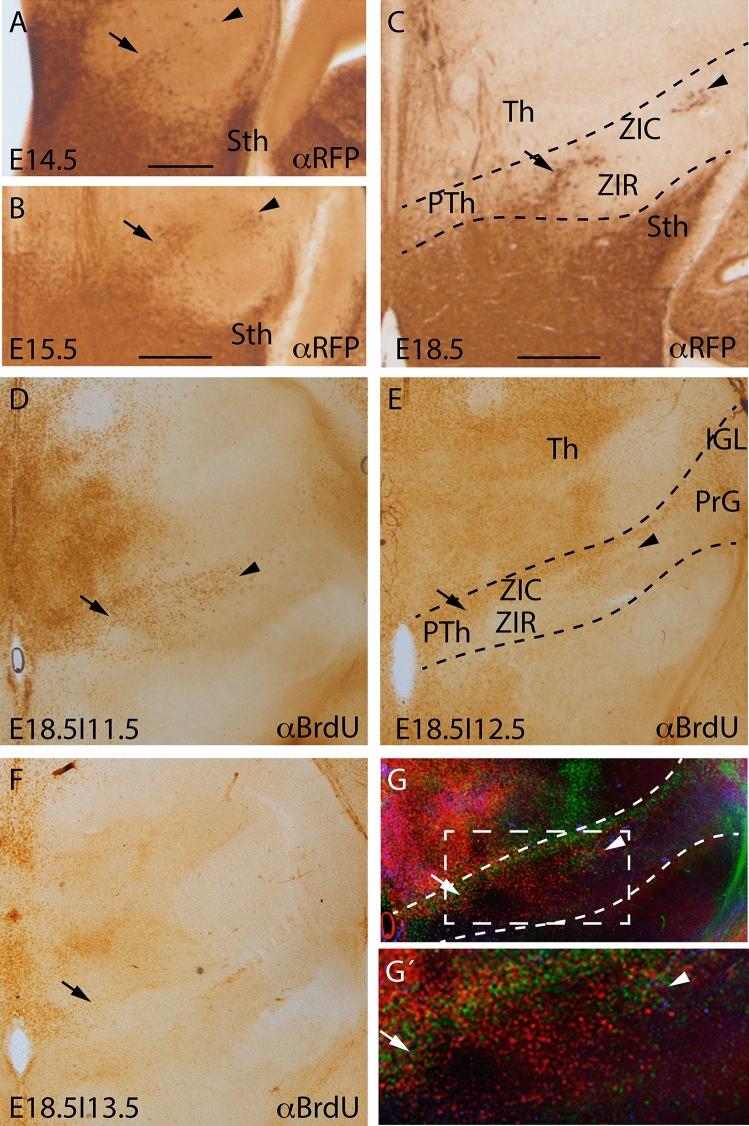
**a–c** RFP immunostaining in frontal brain sections of E14.5 (**a**), E15.5 (**b**) and E18.5 (**c**) *Nkx2.1*^*cre/*+^; *tdTomato*^*flox/*+^ embryonic brains. The arrow indicates the colonization of tomato positive neurons in the zona incerta caudal (prethalamic territory) and the arrowhead the lateral. **d-f** BrdU immunostaining in transversal sections of E18.5 embryonic brain, BrdU injected at E11.5 (**d**), 12.5 (**e**) and 13.5 (**f**). The prethalamic neurons of the zona incerta and reticular nucleus present different born dates. **g** Merge of the photos displayed in **d–f** with red color for E11.5, green color for E12.5 and blue color for E13.5 focused in the area of interest. The dashed box in G indicate the magnified area in G´. The dashed lines delimitate the prethalamic territory. *IGL* intergeniculates nucleus, *PrG* pregeniculate nucleus, *Pth* prehtlamus, *Sth* subthalamic nucleus, *Th* thalamus, *ZIC* zona incertacaudal, *ZIR* zona incerta rostral. Scale bar = 200 μm

We detected the first prethalamic RFP positive cells before E14.5 (arrow in Fig. 5a). At E15.5, the number of positive neurons increased, and in the lateral part, a parch of cells was also identified (arrowhead in Fig. 5b). At the end of the embryonic period, the area of the zona incerta was occupied by *Nkx2.1* derivatives. In the medial part, both rostral and caudal zona incerta (following recent description of the prethalamic territory by Puelles et al. 2020) displayed *Nkx2.1* derivatives, but in the lateral part only the caudal part was invaded (arrow in Fig. 5b). A small patch of positive cells, detected previously at E15.5, was located in the lateral region of this nucleus (arrowhead in Fig. 5c).

It has been described in rat that the zona incerta neurons are born in a two days period (from E13.5 to E15.5; Altman and Bayer 1978). Thus, we decided to study the fate map of the prethalamic precursors along time by the BrdU administration. In mouse embryos, we detected BrdU-positive neurons in the prethalamus mainly between E11.5 and E13.5 (Fig. 5d–g; being G an overlap of the photos displayed in D-F). Scattered positive cells were also detected later on. The medial prethalamic territory, coinciding with the *Nkx2.1* derivatives colonization, is populated by neurons born at E11.5 (Fig. 5d and red color in Fig. 5g, G’). The rostral zona limitans (prethalamic part of the boundary between the rostral diencephalic prosomeres), a periventricular population and intermediate mantle regions of the prethalamus were born at E12.5 (Fig. 5e and green color in Fig. 5g, G’). Finally, the most lateral regions of the prethalamic region displayed mixed populations between E12.5 and E13.5 born neurons (Fig. 5f and blue color in Fig. 5g. G’). Therefore, all together, this late tangential migratory stream that populates the prethalamic area is born at E11.5 but colonize that territory from E13.5 onwards.

### Neuronal characterization of Nkx2.1 migrated derivatives

Since *Nkx2.1*-positive tangential migration in the telencephalon gives rise to interneurons, we hypothesized that these *Nkx2.1* derivatives could also be interneurons. To demonstrate it, we used E18.5 transgenic mouse embryos (*Nkx2.1*^*Cre/*+^*; Tomato*^*flox/*+^*, GAD67*^*GFP*/+^), which expresses the tomato gene under the promoter of *Nkx2.1* and the *GFP* under the promoter of *Gad67*. We performed a double immunofluorescence labelling in red for the RFP and in green for the GFP.

**Fig. 6:**
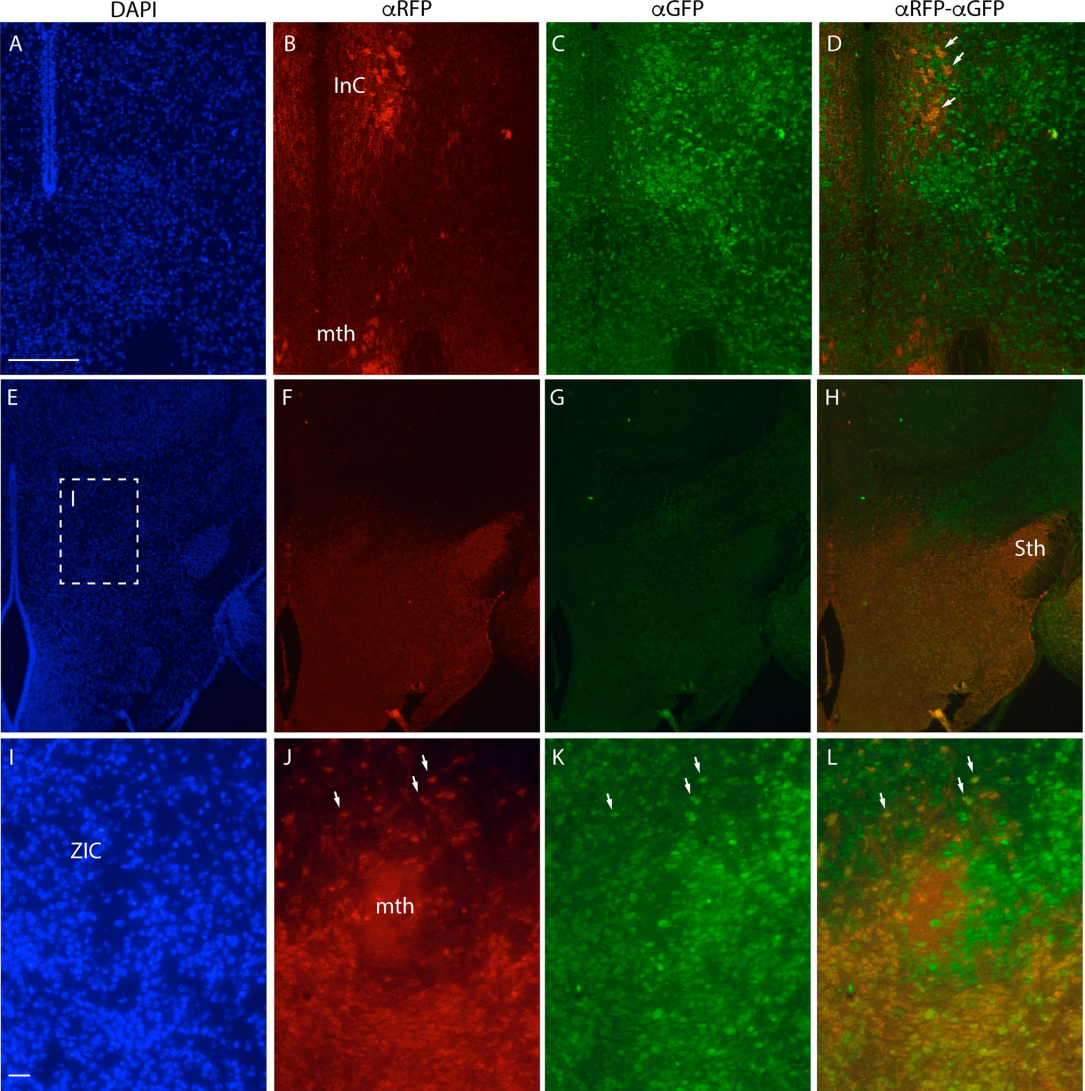
**a**–**c**; **e**–**g**: Double immunostaining against RFP and GFP and DAPI staining of frontal brain section of E18.5 transgenic *Nkx2.1*^*cre/*+^;* tdTomato*^*flox/*+^;* GAD67*^*GFP/*+^. The dashed line box indicates the area displayed in the following images. **i–k** Magnification of the zona incerta area, displaying the mamillothalamic tract. **d**, **h**, **l** Merge of RFP and GFP images; the orange color indicates the tomato positive neurons that display a gabaergic phenotype. The arrows point some examples of gabaergic neurons with a *Nkx2.1* lineage. *InC* Interstitial column, *Sth* Subthalamic nucleus, *mth* mamillothalamic tract, *ZIC* zona incerta caudal. Scale bar = 200 μm in **a–h** and 20 μm in **i–k**

In transversal sections of E18.5 brains (Fig. 6a, e), we observed the final location of the migrated neurons from the early and late streams (Fig. 6b, f). The *Nkx2.1* derivatives from the early migration are located in a periventricular region of the mes-diencephalic basal plate (Fig. 6a–d). This area contained an interstitial column with a spare number of gabaergic neurons and it was integrated into the reticular formation (arrows in Fig. 6d). In a rostral section we observed, apart from the hypothalamic domain, the subthalamic nucleus and the zona incerta strongly populated by *Nkx2.1* cell derivatives (Fig. 6e–h). The subthalamic nucleus, originated in the retromamillar domain was shown (Fig. 6e–h). Finally, the zona incerta caudal showed the partial colonization of the *Nkx2.1* cell derivatives (Fig. 6i–l) together with an interneuronal phenotype (arrows in Fig. 6l).

## Discussion

The expression pattern of transcription factors involved in the determination and differentiation of the neural tissue is not always constant along time. The transcription factor *Nkx2.1* has been generally used as a marker of the terminal and peduncular basal hypothalamus (tuberal, retrotuberal and mamillary region; Puelles and Rubenstein 2015; Puelles 2019), excepting the retromamillary region (see Figs. 8.9 and 8.10 in Puelles et al. 2012). Our results have demonstrated that up to E10.5 embryos this transcript is also expressed in the peduncular hypothalamus (retromamillary region) and, therefore, it is required in early determination of all the hypothalamic territory. It is plausible to hypothesize that the correct differentiation of the peduncular hypothalamus required the *Nkx2.1* switch off (it belongs to a potent repressive transcription factor family; Muhr et al. 2001). The inhibition of this repression would allow the switch on of specific differentiation genetic cascades needed to determine the neuronal populations of this caudal hypothalamic territory.

Well-known tangential migrations events have been described within the hypothalamic territory and from surrounding areas into the hypothalamus. They involve the paraventricular nucleus, the ventromedial nucleus, the ventroposteromedial nucleus, the subthalamic nucleus, and the migrations involving peptidergic neurons (Alvarez-Bolado et al. 2000; Skidmore et al. 2008; Zhao et al. 2008; Morales et al. 2011, 2014; Díaz et al. 2015; Puelles et al. 2012).

**Fig. 7:**
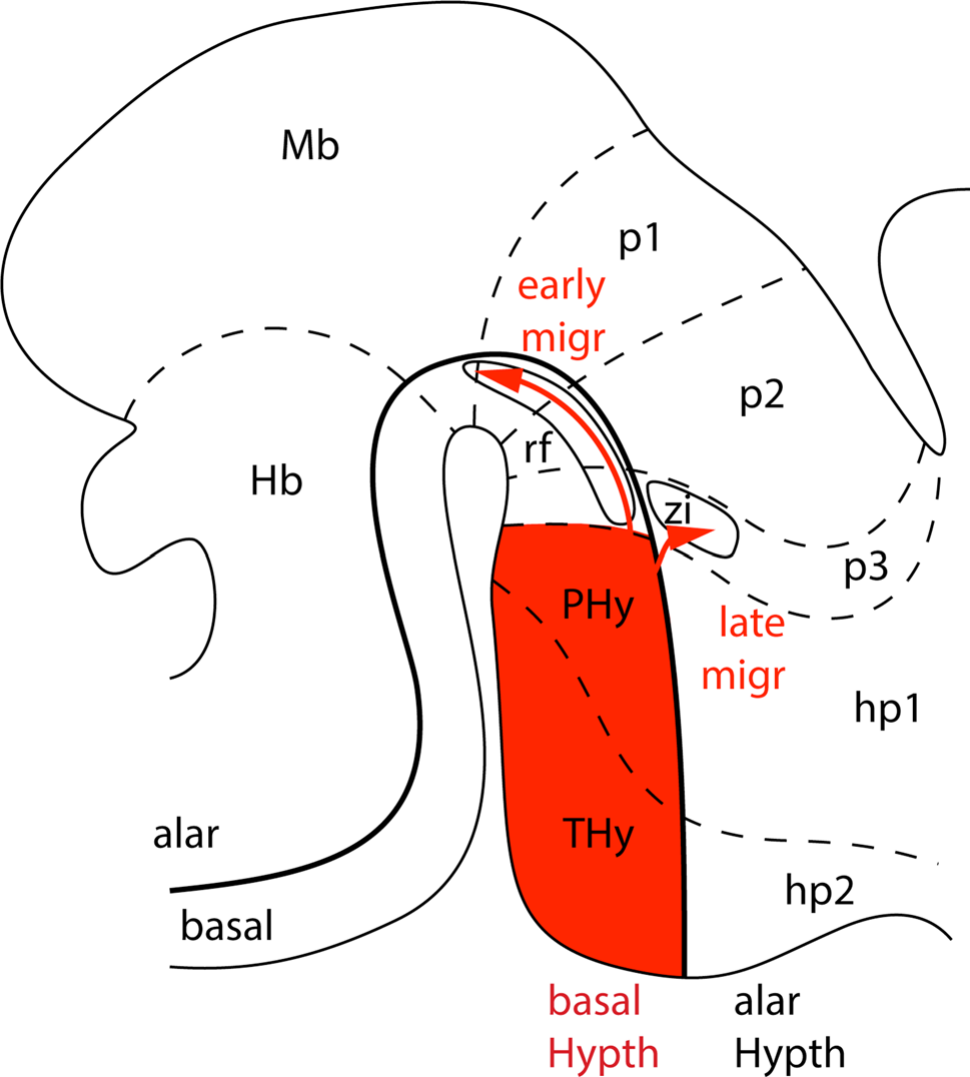
Schematic drawing of the developing brain with the prosomeric model represented. The basal hypothalamic territory in both hypothalamic prosomeres contain the RFP positive area. The hp1 contains the peduncular hypothalamus and the pallial/subpallial telencephalic domains. The hp2 contains the terminal hypothalamus and the preoptic domain. The two migration processes are displayed by red arrows and terminates in the reticular formation and the zona incerta. The alar/basal boundary is indicated by a thick black line. *Hb* hindbrain, *hp1* hypothalamo-telencephalic prosomere 1 (peduncular hypothalamus), *hp2* hypothalamo-telencephalic prosomere 2 (terminal hypothalamus), *Hypth* hypothalamus, *Mb* midbrain, *p1-3* diencephalic prosomeres 1–3, *PHy* peduncular hypothalamus, *rf* reticular formation, *Thy* terminal hypothalamus, *zi* zona incerta

The results of our work have demonstrated for the first time that the NKX2.1 positive basal hypothalamic territory is the source of two tangentially migrated neurons toward other territories (Fig. 7). This property was largely probed by the telencephalic positive domain by fate map analysis (Xu et al. 2008). In the medial ganglionic eminence, the *Nkx2.1* silencing is needed to allow the expression of neuropilins receptors (*Nrp1* and *Nrp2*). Once expressed, the immature neurons are repelled by semaphorins and this repulsion forces them to start their migration. The neurons that retained *Nkx2.1* expression remained in semaphorin-positive territories (Nóbrega-Pereira et al. 2008; Butt et al. 2008; Elias et al. 2008; Kanatani et al. 2015). However, in the hypothalamus, large neuronal populations as the mamillary bodies retain *Nkx2.1* expression and at the same time express high levels of *Nrp1* (Allen Developing Mouse Brain Atlas) and areas as the retromamillary domain silence Nkx2.1 being negative for Nrp1 (Allen Developing Mouse Brain Atlas). Therefore, the *Nkx2.1* silencing must involve the activation of different neuronal migration signaling mechanisms.

The two tangential migration described in the present work display different timing and direction. The early migration moves toward caudal positions, whereas the late migration travels to caudodorsal areas (Fig. 7).

On the one hand, the early stream of *Nkx2.1* neuronal derivatives detected migrated into the basal dien-mesencephalon. They were located close to the periventricular gray in a longitudinal column belonging to the reticular formation. This column has been identified as the interstitial column (Moreno-Bravo et al. 2010, 2012). Other members of the Nkx family, such as *Nkx6.1* and *Nkx6.2*, also contribute with derivatives to this neuronal formation (Moreno-Bravo et al. 2010). The interneurons are integrated into a functional pre-oculomotor formation related with the saccadic movements of the eyes.

On the other hand, the late stream colonizes the alar prethalamic domain. We detected a group of positive cells in the periventricular territory of the zona incerta complex (both rostral and caudal). In the intermediate territory of these neuronal populations, the *Nkx2.1* derivatives are concentrated in the caudal part of the zona incerta. An isolated group is located in the lateral portion of the zona incerta caudal. Based on the recent genoarchitectural description of the prethalamic territory by Puelles et al. (2020), the zona incerta is an heterogeneus gabaergic territory with a diverse group of neurons (cytoarchitectonically and by chemical nature). It is connected with the thalamus, hypothalamus, brainstem and spinal cord (Mitrofanis 2005). Therefore, to obtain the needed diversity of gabaergic neuronal subtypes, this neuronal population receives contributions also, as we have demonstrated, by the basal hypothalamic territory. This diversity of origins has been also illustrated by the localization of the proteolipid protein (*Plp*) diencephalic cells; they also contribute with gabaergic neurons to the zona incerta (Delaunay et al. 2009).

Moreover, it is well accepted that the hypothalamic territory receives and contain diverse tangential migratory events. In our work, we have demonstrated two tangential migratory streams originated in the hypothalamus that populate mainly diencephalic territories.

## Materials and methods

### Mouse strains

The three mouse lines used and the genotyping have been already described: *Nkx2.1*^*cre/*+^ (Marín et al. 2000) R26R*-CAG-TdTomato* from Jackson Laboratories (strain 007905) and *GAD67*^*GFP/*+^ (Tamamaki et al. 2003). The mouse embryos examined were of two types: *Nkx2.1*^*cre*/+^; *tdTomato*^*flox*±^, generated by crossing heterozygous mouse males (*Nkx2.1*^*cre/*+^) with homozygous reporter females (*tdTomato*^*flox/flox*^); and *Nkx2.1*^*cre*/+^; *tdTomato*^*flox/*+^; *Gad67*^*gfp/*+^, generated by crossing a double heterozygous male (*Nkx2.1*^*cre/*+^; *tdTomato*^*flox/*+^) with homozygous female (*Gad6*^*GFP/*+^).

For staging, the day of vaginal plug was counted as embryonic day 0.5 (E0.5). For immunochemistry, embryos were fixed in 4% paraformaldehyde in PBS overnight and completely dehydrated for storage at − 20 ℃.

All mouse experiments were performed according to protocols approved by the Universidad Miguel Hernandez OEP committee.

### Immunohistochemistry

Samples were paraffin-embedded and sectioned at 10 µm. Sections were dewaxed and rehydrated. Then, the tissue was incubated with hydrogen peroxide (H_2_O_2_) at 0.9% for 30 min to inactivate the endogenous peroxidase activity. Afterwards, the sections were washed three times in PBS-T (phosphate buffer solution with 0.1% triton) and boiled in 0.01 M sodium citrate. Then, the tissue was blocked during 1 h with blocking solution (PBS-T, 0.1% albumin bovine serum, 10% lysine and 0.01% Azide) and incubated overnight at room temperature with primary antibodies diluted in blocking buffer solution. After that, the tissue was washed three times in PBS-T.

For the colorimetric immunohistochemistry, samples were incubated 1 h with the appropriate biotinylated antibody diluted at 1:200 in PBS-T, washed three times with PBS-T, incubated with avidin–biotin complex diluted at 1:500 in PBS-T and washed again in PBS. Finally, tissue was incubated in a PBS 1:100 DAB (Diaminobenzidine) and 0.003% H_2_O_2_ and washed with PBS to stop the reaction.

For double immunohistochemistry, the samples were incubated in the appropriate fluorochrome conjugated secondary antibodies diluted at 1:500 in PBS-T and washed with PBS. Finally, the tissue was incubated in DAPI diluted at 1:1000 in PBS and washed with PBS.

Primary antibodies used were: α-RFP rabbit polyclonal IgG (PM005 MBK, 1:500), α-NKX2.1 (TTF1) rabbit polyclonal IgG (PA 0100 Biopat, 1:1000) and α-GFP chicken polyclonal IgG (A-11122 Molecular Probes, 1:500) and α-BrdU mouse monoclonal IgG (M0744 Dako, 1:200). The secondary antibodies used were: α-Rabbit IgG (BA-1000 Vector), α-Mouse IgG (BA-9200 Vector), α-Rabbit IgG, Alexa Fluor 594 (A 21,207 Molecular Probes) and α-Chicken IgY (IgG) FITC (F 8888 Sigma).

### Birth dating by BrdU labeling

For detection of the peak of neurogenic proliferation, BrdU was administered intraperitoneally to the pregnant females (3 mg/100 g body weight) every 2 h, for a period of 10 h (five injections in total) starting at desired stages. The embryos were extracted at E18.5.

### Microscopy

Images were taken with a camera associated to the stereomicroscope (Leica Fluo-III) and the bright field or immunofluorescence images were taken with a camera (Leica DFC500) associated to the stereomicroscope. The images and figures were made with Adobe system.

### Timelapse

For the time-lapse experiments, the embryos were extracted and dissected in cold PBS. Organotypic Neural Tissue explant Cultures (ONTC) were performed as described in (Echevarría et al. 2001). The ONTCs were placed in a polycarbonate membrane (MilliCell PICMORG50) with neurobasal medium and incubated during the experiment at 37 ℃, and 5% CO_2_.

A TCS-SP2-AOBS laser scanning spectral inverted confocal microscope (fitted with temperature and CO_2_ control; Leica Microsystems) was used for live imaging of the ONTC. Images were collected every 20 min during 18 h. All the focal planes were merged to visualize the maximum projection. Videos were processed with ImageJ (FIJI) software.

## Electronic supplementary material

Below is the link to the electronic supplementary material.

Supplementary ESM1. The time-lapse video showed the red fluorescence neurons that previously expressed *Nkx2.1* (now tomato) migrating tangentially towards caudo-dorsal territories in a *Nkx2.1cre/+; tdTomatoflox/+* ONTC. The immature neurons migrate toward caudal positions into the mes-diencephalic tegmentum (MP4 7273 KB)

## Data Availability

All material and data used are available upon request.
